# Comprehensive Identification of *Salmonella enterica* Serovar Typhimurium Genes Required for Infection of BALB/c Mice

**DOI:** 10.1371/journal.ppat.1000529

**Published:** 2009-07-31

**Authors:** Roy R. Chaudhuri, Sarah E. Peters, Stephen J. Pleasance, Helen Northen, Chrissie Willers, Gavin K. Paterson, Danielle B. Cone, Andrew G. Allen, Paul J. Owen, Gil Shalom, Dov J. Stekel, Ian G. Charles, Duncan J. Maskell

**Affiliations:** 1 Department of Veterinary Medicine, University of Cambridge, Cambridge, United Kingdom; 2 Arrow Therapeutics Ltd., London, United Kingdom; 3 Centre for Systems Biology, School of Biosciences, University of Birmingham, Edgbaston, Birmingham, United Kingdom; 4 Department of Molecular Biology and Biotechnology, University of Sheffield, Sheffield, United Kingdom; The Rockefeller University, United States of America

## Abstract

Genes required for infection of mice by *Salmonella* Typhimurium can be identified by the interrogation of random transposon mutant libraries for mutants that cannot survive *in vivo*. Inactivation of such genes produces attenuated *S.* Typhimurium strains that have potential for use as live attenuated vaccines. A quantitative screen, Transposon Mediated Differential Hybridisation (TMDH), has been developed that identifies those members of a large library of transposon mutants that are attenuated. TMDH employs custom transposons with outward-facing T7 and SP6 promoters. Fluorescently-labelled transcripts from the promoters are hybridised to whole-genome tiling microarrays, to allow the position of the transposon insertions to be determined. Comparison of microarray data from the mutant library grown *in vitro* (input) with equivalent data produced after passage of the library through mice (output) enables an attenuation score to be determined for each transposon mutant. These scores are significantly correlated with bacterial counts obtained during infection of mice using mutants with individual defined deletions of the same genes. Defined deletion mutants of several novel targets identified in the TMDH screen are effective live vaccines.

## Author Summary

*Salmonella* Typhimurium infection of mice is an established model of systemic typhoid fever in humans. Mutations that inactivate genes that are important for virulence produce attenuated *S.* Typhimurium bacteria that have potential for use as live vaccines. To investigate the infection process we have produced a large pool of random insertion mutants, and developed a novel microarray-based technology, Transposon Mediated Differential Hybridisation (TMDH), that allows us to determine the gene disrupted by each insertion. Comparison of data obtained from the mutant pool grown in laboratory culture (input) with equivalent data produced after passage of the pool through mice (output) enables genes that are important for the infection process to be determined, since they are absent or less prevalent in the output pool. We have constructed defined deletion mutants of several of the candidate genes identified in the TMDH screen, and have shown that they are attenuated for virulence and effective live vaccines.

## Introduction

*Salmonella enterica* serovar Typhimurium (*S.* Typhimurium) infection of mice is well established as a model of systemic typhoid fever in humans [1]. The system is particularly useful for identifying the genetic determinants of *Salmonella* virulence, usually by comparing the *in vivo* growth of mutants with their wild type parents. If a mutant is unable to survive in the mouse model it is inferred that the disrupted gene is important for infection. Several such mutants have been used to induce an immune response that is protective against subsequent infection with wild type *S.* Typhimurium, and have subsequently been translated for use as live attenuated vaccines in humans and food-producing animals [2].

Most often, the mouse model has been used to investigate *Salmonella* infection on a gene-by-gene basis. However, recent developments in molecular biology allow pools of many mutants to be screened in parallel. The first example of this was signature-tagged mutagenesis (STM), which employs transposons containing unique sequence tags that can be amplified by PCR and identified by Southern hybridisation. These transposons are used to generate mixed pools of mutants, which are used to infect an animal. Bacterial DNA recovered from the animal can be interrogated for the presence of the tags, and compared to similar data obtained from the inoculum to identify attenuated mutants that do not survive *in vivo*. This method was developed in a study of *S.* Typhimurium in the mouse model of typhoid fever [3], using input pools of 96 tagged mutants per animal. This led to the discovery of *Salmonella* pathogenicity island 2 (SPI-2), which encodes a type III secretion system (T3SS) that is critical for systemic infection and intracellular pathogenesis [4]. The island was so named to distinguish it from the well-characterised SPI-1, which also encodes a T3SS that is important in intestinal adhesion and invasion [5]. Morgan *et al.* [6] used STM to identify *S.* Typhimurium genes required for colonisation of calves and chicks, using the same STM mutant library to infect the different hosts, hence enabling comparison of the gene sets required for infection of different livestock species. This study showed that SPI-1 and SPI-2 mutants that were defective in colonization of calves were able to colonize chicks and led to further characterisation of a SPI-4 locus, required for infection of calves, but which appeared to be less important in the intestinal colonization of pigs in a further STM screen with this library [7].

There are various limitations associated with STM, including the size of mutant pools given the number of unique tag sequences that it is practical to use, and the fact that STM may not lead to the identification of mutations that cause only a small reduction in fitness to survive within a host [8]. An alternative approach employs DNA microarray technology to screen a library of transposon mutants. The parallel nature of microarrays allows the simultaneous screening of much larger pools of mutants than is possible using STM. The use of a custom transposon containing an outward facing promoter allows the *in vitro* production of labelled transcripts that are homologous to the regions flanking the sites of transposon insertion. These can be used to hybridise to the microarray, bypassing the need for a PCR amplification step that can lead to non-reproducible results [9].

Microarray-based screening approaches have recently been applied to the study of *S.* Typhimurium infection of mice. Using such methods, Chan *et al*. [10] identified *S.* Typhimurium mutants that cannot survive in RAW 264.7 macrophage-like cells. Within the population of negatively selected mutants there was a significant overrepresentation of genes located in SPI-1 (36/395) and SPI-2 (51/395) indicating their importance in this infection model. Screening the same transposon library through BALB/cJ mice similarly revealed significant overrepresentation in the negatively selected population of SPI-2 (27/187) and genes involved in lipopolysaccharide (LPS) biosynthesis (9/187), with an overrepresentation of SPI-1 genes (5/187) that fell marginally short of significance at the 5% level. In a follow-up study, Lawley *et al*. [11] used similar methods to study genes required by *S.* Typhimurium to survive for up to 28 days in the spleens and livers of *Nramp1* ^r^ mice, a model of the carrier state in human typhoid fever. In this model genes from SPI-1 to SPI-6 were important for systemic infection. The involvement of SPI-1 was a novel finding since that island had previously been thought to be involved only in the gastrointestinal phase of infection. It is possible that SPI-1 genes may be required for *S.* Typhimurium to re-establish intracellular growth during persistent infection. Other genes important for long-term systemic infection included LPS genes, fimbrial genes, and horizontally acquired genes on the virulence plasmid and within prophage elements.

Here we describe transposon mediated differential hybridisation (TMDH) [12], and its application for the simultaneous, genome-wide identification of genes required for growth of *S.* Typhimurium in an acute infection of BALB/c mice over 2 days. Our method improves significantly on earlier studies through the use of high-density tiling microarrays and a novel bioinformatics algorithm to determine the positions of transposon insertions with a high degree of accuracy. This has allowed the unambiguous identification of transposon mutants within 2824 *S.* Typhimurium genes. Many of these are attenuated in the mouse typhoid model, and may encode potential targets for the development of novel antimicrobial therapeutics. We also show that defined deletion mutants of some of the targets identified in the TMDH screen can act as novel live-attenuated vaccines that protect against subsequent challenge with wild-type *S.* Typhimurium. The technology we present here is generic and can be applied to any pathogen for which there is a genome sequence, a method of generating large numbers of insertion mutants (in this case, transposition) and a model of infection in which to identify mutants that are attenuated for virulence.

## Materials and Methods

### Transposon-Mediated Differential Hybridisation (TMDH)

**Figure 1:**
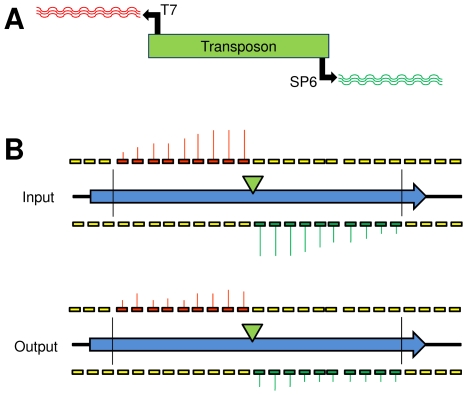
Diagrammatic representation of the TMDH process. (A) A library of transposon mutants is obtained using a custom transposon with outward-facing T7 and SP6 promoters. Genomic DNA is extracted from the library and digested using a restriction endonuclease (*Rsa*I). Labelled RNA run-offs are obtained from the T7 and SP6 promoters by *in vitro* transcription. (B) The labelled RNA run-offs are hybridised to genome-wide tiling microarrays. By examining the distribution of microarray signals between *Rsa*I restriction sites (vertical black lines) it is possible to infer the location of the transposon (green triangle). Comparison of the library grown *in vitro* (input) with the library obtained after passage through a mouse (output) allows attenuating mutants to be identified, since they give lower signals in the output.

The TMDH procedure is outlined in Figure 1. A library of around 10,000 transposon mutants was generated using custom Tn5 and Mu transposons, containing outward-facing T7 and SP6 promoters. Genomic DNA was isolated from the library, and *in vitro* transcription was induced from the T7 and SP6 promoters in the presence of fluorescently-labelled dNTPs. DNaseI was used to remove the genomic DNA, leaving labelled RNA run-offs, which were hybridised to high-density tiling microarrays. Analysis of the microarray data allows the genomic position of the transposon insertions to be determined. This process was performed for the original library (input), and for mutant pools recovered from the livers of duplicate intravenously (i.v.) infected mice (output). Mutants that are present in the input pool but which are absent or less prevalent in the output pools are inferred to be attenuated *in vivo*.

### Construction of Tn5 and Mu TMDH transposons for use in *S.* Typhimurium

TMDH transposons were based on Tn5 and Mu transposasome constructs from Epicentre (EZ:Tn5 R6Kγori/KAN-2 Transposon and HyperMu R6Kγori/KAN-1 Transposon). The constructs were adapted for use in TMDH by the addition of outward-facing T7 and SP6 promoters, which allow the generation of both left- and right-arm RNA products corresponding to the regions flanking the site of transposon integration in genomic DNA [13], and incorporation of homing endonuclease recognition sites for I-*Sce-I* and PI-*Psp-I*. These are rare-cutting enzymes and incorporation of their recognition sites into the transposons permits the introduction of the sites into a bacterial genome following transposition. Following digestion of transposed genomic DNA with the rare-cutting enzyme, a ligation-capture method can be carried out to rescue transposon ends and obtain flanking sequence data (see below). The Tn5 and Mu TMDH transposons were cloned into pBAD-TOPO (Invitrogen Life Technologies) and transformed into TOP10 *E. coli* chemically competent cells according to the Invitrogen protocol, with selection on LB agar (Sigma-Aldrich) plates supplemented with 30 µg/ml kanamycin (Sigma-Aldrich). Colonies were picked and grown in L-Broth (Sigma-Aldrich) and plasmids were isolated using a Qiagen QIAprep spin miniprep kit. The Tn5 and Mu constructs were sequenced and have been deposited into the EMBL database (accession numbers AX828670 and FN394965). The plasmids were transformed into electrocompetent *S.* Typhimurium SL1344 with selection on 30 µg/ml kanamycin. Single colonies were picked and grown up further in order to purify the plasmids using a Qiagen QIAfilter Mega Plasmid Prep, which produced 2 mg of plasmid in 2 ml of TE (pH 8.5).

### Construction and characterization of transposon libraries in *S.* Typhimurium SL1344

Tn5 and Mu TMDH libraries of around 5,000 mutants each were generated in *S.* Typhimurium SL1344. 100 µg of Tn5 or Mu pBAD-TOPO TMDH constructs were digested overnight at 37°C with *Xmn*I and *Nco*I (New England Biolabs) in NEB buffer 2 and BSA in a final volume of 200 µl. The entire digest was then run out on a 0.8% agarose gel, the insert bands were cut out and the DNA was extracted from the gel using the Qiagen QIAquick Gel Extraction kit and eluted in 50 µl TE (pH 8.5). The purified Tn5 and Mu transposon DNA was used in the Epicentre *in vivo* transposition protocol using EZ-Tn5 Transposase or HyperMu™ MuA Transposase as appropriate. Transposon DNA was added to transposase and glycerol in a 1∶2∶1 ratio and incubated at room temperature for 1 h. The transposasome complex was then electroporated into electrocompetent SL1344 cells using a 2 mm cuvette, at 200 Ω, 25 mF and 12 kV/cm. Recovery was in 1 ml SOC medium (Gibco) at 37°C for 1 h then the bacteria were plated out on Tryptic Soy Agar (TSA, Oxoid), supplemented with 50 µg/ml kanamycin, and grown overnight at 37°C. Colonies were picked and grown overnight at 37°C in 0.5 ml L-Broth supplemented with 25 µg/ml kanamycin, in 2 ml 96-well blocks (Fisher Scientific). Overnight cultures of 5,184 Tn5 and 5,184 Mu TMDH transposon mutants were stored at −80°C in 20% glycerol in L-Broth in 96-well microtitre plates.

96 of the Tn5 mutants were streaked on LB agar plates supplemented with 100 µg/ml ampicillin to check for sensitivity. All were sensitive, showing that there had been no carry-through of undigested pBAD-TOPO construct into the gel-purified transposon DNA. Tn5 mutants 1 to 50 were screened on microscope slides for agglutination with anti-O4 antiserum (Remel Europe Ltd.). All mutants agglutinated with the antiserum indicating the bacteria expressed an intact O-antigen following electroporation.

### The *in vivo* TMDH procedure: mouse model of infection

To prepare inocula for *in vivo* TMDH in mice, 5,184 Tn5 and 5,184 Mu TMDH transposon mutants were grown up individually in 1 ml L-Broth in 2 ml 96-well blocks, at 37°C overnight. Cultures were combined into 20 pools of 480 mutants and 2 pools of 384 mutants for inoculation into mice. 3 ml of each pooled culture was removed for measurement of OD_600_ in order to estimate bacterial numbers. The remainder was used for preparation of input-pool genomic DNA (see below). Cells from 3 ml cultures were recovered by centrifugation (4300×*g* for 10 min), resuspended in 3 ml phosphate buffered saline, pH 7.5 (PBS) and diluted to give an appropriate number of bacteria for the inoculum. An aliquot of the inoculum was plated on LB agar in triplicate in order to obtain an accurate viable count. A dose of 10^6^ colony-forming units (CFU) was chosen empirically as sufficient to prevent random dropout of mutants, and was inoculated in 0.2 ml PBS into the tail veins of duplicate six- to eight-week-old BALB/c mice (Harlan). All animal procedures were carried out in accordance with the Animals (Scientific Procedures) Act (1986).

### Recovery of organs from infected mice

Spleens and livers were recovered 2 days post-infection and homogenised in 10 ml distilled water, then 100 µl aliquots were plated for viable counts on LB agar. The remainder of the homogenate was plated on three 50 ml LB agar plates and incubated at 37°C overnight.

### Preparation of genomic DNA

For each pool of mutants, genomic DNA from the input pool and the two liver output pools was prepared. For the input pools, bacteria were collected from 200 ml of pooled overnight culture by centrifugation in a benchtop centrifuge at 4300×*g* at 15°C for 10 min. For the output pools, bacteria from confluent plates were harvested by adding 10 ml of L-Broth to each plate, then bacterial suspensions were pooled and vortexed, and cells from a 20 ml aliquot were recovered by centrifugation at 4300×*g* for 10 min. In each case, the pellet was resuspended in 20 ml of Tris (10 mM, pH 8) EDTA (10 mM) (TE), then 400 µl of 10 mg/ml lysozyme (Sigma-Aldrich) in water was added and incubated at 42°C for 30 min. 200 µl of Qiagen Proteinase K, 40 µl of Qiagen RNaseA, and 2 ml of 10% (w/v) N-lauryl sarcosine (Sigma-Aldrich) were added and incubated for 1 hr (input pools) or 2 hr (output pools), or until completely lysed (clear) at 50–55°C. Lysates were extracted by adding 1 volume of buffered phenol (Sigma-Aldrich), shaking, and centrifuging at 7000×*g* for 10 min. The aqueous layer was removed and extracted with 1 volume of phenol∶chloroform∶isoamyl alcohol (IAA) (25∶24∶1), then once more with chloroform∶IAA (24∶1). DNA was precipitated with 2 volumes of ethanol, recovered, washed in 70% (v/v) ethanol, then transferred to a 1.5 ml tube containing 0.5 ml of TE and left at 4°C to dissolve. The DNA concentration and A_260_/A_280_ ratio were measured on a NanoDrop 1000 spectrophotometer (Thermo Scientific). Genomic DNA was also prepared similarly from a 50 ml wild-type *S.* Typhimurium SL1344 culture grown overnight at 37°C in L-Broth for use as an untransposed control (see below).

### Restriction digests and *in vitro* transcription

10 µg of genomic DNA from each TMDH input and output pool, and from the untransposed control DNA, was digested using *Rsa*I (Promega) overnight at 37°C. Digested DNA was cleaned using a Qiagen QIAquick PCR Purification Kit and eluted in 30 µl RNase-free water (Qiagen). The DNA concentration and A_260_/A_280_ ratio were measured on a NanoDrop 1000 spectrophotometer. Equal amounts of digested DNA from 2 or 3 pools of mutants were combined to give 8 sets of 960–1440 mutants each for microarray analysis (see Table 1). *In vitro* transcription (IVT) was induced from the transposon T7 and SP6 promoters using 500 ng of genomic DNA in a 20 µl MEGAscript T7 Kit or MEGAscript SP6 Kit reaction (Ambion Inc.), with half the UTP replaced with 5-(3-aminoallyl)-UTP (aa-UTP; Ambion Inc.). RNA run-offs were treated with TURBO DNase (Ambion) and purified on Qiagen RNeasy MinElute columns. Purified RNA from input or output pools was post-labelled with Cy5 and RNA from wild-type (untransposed) control DNA with Cy3, using CyDye Post-Labelling Reactive Dye Packs (GE Healthcare), and the reactions were stopped with 4 M hydroxylamine (Sigma-Aldrich). The resultant labelled RNA was purified again on Qiagen RNeasy MinElute columns, and used for hybridization to DNA microarrays.

**Table 1:** Number of mutants investigated in each array set.

Array Set	Number of Mutants
Set1	960 (2×480)
Set2	960 (2×480)
Set3	1440 (3×480)
Set4	1440 (3×480)
Set5	1440 (3×480)
Set6	1440 (3×480)
Set7	1440 (3×480)
Set8	1248 (1×480, 2×384)

In parentheses are the number and size of the mutant pools used to infect individual mice. These were pooled as indicated for the microarray experiments.

### Microarray methods

A set of 60-mer oligonucleotide probes was designed based on the *S.* Typhimurium LT2 genome sequence (accession numbers AE006468 and AE006471). The probes were spaced approximately every 100 bases on both strands across the whole genome, with the exception of repetitive regions for which unique probes could not be designed. As a different *S.* Typhimurium strain, SL1344, was used for the TMDH experiment it was necessary to identify the positions of the SL1344 genome that corresponded to each probe. The SL1344 genome sequence and preliminary annotation was obtained from the Wellcome Trust Sanger Institute (http://www.sanger.ac.uk/Projects/Salmonella). Each microarray probe sequence was used as the query in a blastn search of the SL1344 genome sequence, and was considered to match uniquely if the top hit showed >80% identity over >50% of the length of the probe, and the second hit did not fulfil both those criteria. Uniquely matching probes were used in the TMDH analysis, with their hybridization position on the SL1344 genome determined from the BLAST result. Any probes that contained an *Rsa*I restriction site within the central 30 bases were omitted from the analysis, since the probe signal could reflect transcript from either side of the restriction site.

Custom microarrays with 2×105K features per slide were obtained from Agilent Technologies. Each Cy5-labelled RNA run-off from an input or output pool was combined with an aliquot of Cy3-labelled control RNA generated from the same promoter, and fragmented at 60°C for 30 minutes using Agilent Fragmentation Reagent. Fragmented RNA was then hybridised to microarrays in Agilent Gene Expression HI-RPM hybridisation buffer at 65°C for 17 hours in Agilent hybridisation chambers and backings in an Agilent hybridisation oven. Following hybridisation, the arrays were washed with Agilent wash buffers 1 and 2 according to the manufacturer's instructions, followed by one wash in acetonitrile (Sigma-Aldrich) and drying in Agilent Stabilisation and Drying Solution. The arrays were scanned using an Agilent G2565BA scanner using an extended dynamic range of 10% and 100% PMT, and the images analyzed using the Agilent Feature Extractor software version 9.3.5.1. The raw microarray data were uploaded to ArrayExpress (accession number E-MEXP-2076).

### Experimental design and microarray analysis

A separate microarray experiment was performed for each set of 960–1440 mutants. Each experiment consisted of six microarrays, one each for the T7 and SP6 RNA run-offs from the input pool and from the two biological replicate output pools. The IVT product from each mutant pool was labelled with Cy5 and hybridised to the microarray together with the Cy3 labelled IVT product from an untransposed control strain. The untransposed control has two purposes: it allows normalisation of the T7 and SP6 signals without any problems associated with dye bias, and acts as a control to allow identification of the sites of transposon insertion in the input pool.

Microarray data were imported into R [14] using the Bioconductor package Limma [15]. The raw signals were normalised to account for sequence-dependent variation using the Naef and Magnasco method [16],[17], implemented in R by Royce *et al.* [18]. Briefly, this procedure uses a linear model to predict the contribution to the probe signal intensity of each possible nucleotide at each position in the probe sequence. A predicted log signal intensity is computed for each probe based on the model, and subtracted from the observed log signal intensity.

**Figure 2:**
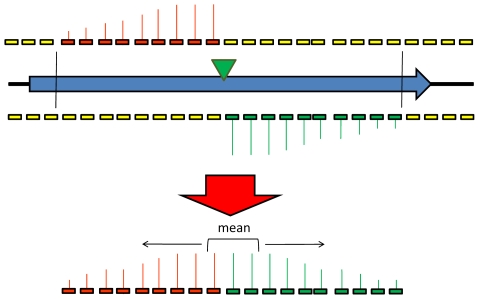
Diagrammatic representation of the TMDH scoring system. The most likely position of the transposon is inferred from the microarray data, and the probes that would detect the signals from a transposon in that position are identified. To summarize the signals of these probes into a single data point, a sliding window approach is used. The geometric mean of the signals is determined for each consecutive set of 3 probes. The highest such value is used to represent the signal for that restriction fragment.

Since the transcripts from each transposon promoter are likely to influence the signal from multiple probes, the individual probe signals cannot be treated as statistically independent. To circumvent this problem the data from all the probes within a single *Rsa*I restriction fragment were summarised to give a single data point. This procedure is illustrated in Figure 2. A single transposon insertion within a restriction fragment is expected to produce transcripts that will hybridise to probes 5′ from the transposon on both strands (“on” probes). All other probes in the restriction fragment should produce only a background signal (“off probes”). The most likely position of a transposon is therefore determined as the position where the sum of the signals from the “on” probes minus the sum of the “off” probe signals is maximal, and the summary score is calculated based on the “on” probe signals. For large restriction fragments it is not appropriate to use an average of all these signals as a summary score, since signal intensities tend to decrease for probes that are distant from the transposon. A sliding window approach was used, with the geometric mean of the signals calculated for each window of three consecutive probes. The highest value was used as the summary score for that restriction fragment.

The situation is complicated somewhat if more than one transposon is present within a single restriction fragment within the same array set. It is possible to distinguish between two inserts within the same fragment if they are in opposite orientations, since the transcripts that correspond to the same strand will be derived from different promoters and hence will be detected on different arrays and not interfere with each other. For this reason each restriction fragment is assigned two summary scores, one for each possible transposon orientation. If multiple transposons are present in the same orientation then the signals will interfere, and it may be impossible to determine the location of each insert. However, since no more than 1440 mutants were investigated on the same array, this is unlikely to represent a significant problem for the analysis of our data.

**Figure 3:**
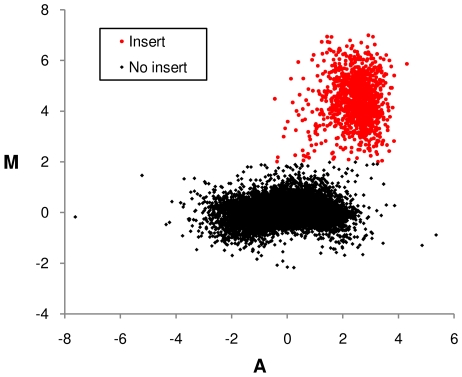
Sample MA plot comparing microarray signals derived from transposed and untransposed DNA. Plot shows log_2_ signal intensity ratio (M) against log_2_ average signal intensity (A) for the summary scores derived from RNA run-offs obtained from transposed and untransposed DNA. An M value of 2 was used as the cut-off to infer the presence of a transposon within a restriction fragment.

To determine the position of the transposon insertions present in the input pool of each set of mutants, summary scores were calculated for the Cy5 signals from the Input T7 and SP6 arrays, and separately for the Cy3 (control) signals from the same arrays. The summary scores are analogous to data derived from a traditional expression microarray, and can be analysed in a similar manner. To do this, the input and control summary scores were converted to the red and green signals of a Limma RGList object [15]. The signals were normalised using a loess curve, and displayed on a plot of log_2_ signal intensity ratio (M) against log_2_ average signal intensity (A; see Figure 3). An M cut-off value of 2 was used to identify summary scores likely to correspond to transposon insertions.

To compare the input and output pools, a similar procedure was followed. In this case the untransposed control signals were used as a standard reference to normalise between arrays, and the ratio of sample:reference signal for each probe was used to calculate the summary scores. This was done separately for the input and two output pools, and the resulting scores analysed in the same manner as single-colour Affymetrix data in Limma [15]. The scores were normalised using a loess curve, as implemented in the Bioconductor package affy [19], and log_2_ fold change (logFC) and P-values were calculated using the linear models implemented in Limma, applying the method of Benjamini and Hochberg [20] to account for multiple testing. These logFC values were normalised between array sets by dividing by the median absolute deviation. The normalised logFC scores and P-values from each set were combined and used to assess the *in vivo* survival of each of the mutants previously identified within the input pool (see Table S1).

### Experimental verification of transposon insertion locations

As part of the TMDH development process, 50 Tn5 mutant colonies were selected from Set6 for DNA sequencing-based verification of the position of the transposon insertion using a ligation capture procedure. The mutants were grown up individually in 2 ml TSB (Oxoid) in the presence of 50 µg/ml kanamycin overnight at 37°C and 200 rpm. 1.5 ml of each was used to prepare chromosomal DNA using the Qiagen DNeasy tissue kit. 5 µl of each of the fifty chromosomal preps was digested with *Eco*RV (NEB) in a final volume of 20 µl, overnight at 37°C. The *Eco*RV was heat inactivated at 80°C for 20 minutes. The 20 µl digest was then re-ligated in 100 µl final volume using Gibco T4 DNA ligase for 48 hours at 4°C. Each re-ligation was then individually cleaned up using a Qiagen gel extraction spin column and eluted in 50 µl water. 4 µl of cleaned up, re-ligated, *Eco*RV-digested chromosomal DNA from each mutant was electroporated into 40 µl of electrocompetent *pir* ^+^ cells using a 1 mm cuvette, 100 Ω, 25 µF and 20 kV/cm, outgrowth was in 1 ml SOC medium (Gibco) at 37°C for 1 h, each of the 50 transformations was then recovered by centrifugation and the pellet resuspended in 50 µl L-Broth and plated out on LB agar supplemented with 30 µg/ml kanamycin, and incubated overnight at 37°C. From each plate where colonies grew, one colony was picked and grown up in 5 ml L-Broth plus 30 µg/ml kanamycin at 37°C overnight. 46 Qiagen minipreps were carried out and 10 µl plasmid DNA was sequenced according to the Beckman CEQ protocol using a transposon-specific primer. *Salmonella* sequence data was obtained for 37 of the mutants.

Additionally, 13 transposon mutants (see Table S2) were selected from Set1 and Set3 based on the results of the microarray analysis. For each mutant, the region flanking the transposon was amplified by PCR, using a transposon-specific primer and a gene-specific primer. The gene-specific primer was designed to anneal ∼200 bp from the approximate position of the transposon as predicted during the automated microarray analysis. PCR was performed using the Expand High Fidelity PCR System (Roche) on a Biometra T3000 thermocycler. Reactions contained 0.5 mM dNTPs (Bioline), 1 µM each primer (Sigma-Genosys), 100 ng template DNA, 1× Expand PCR buffer and 0.75 µl of Expand polymerase mix in a total volume of 50 µl. Bands matching the expected products were excised from a 0.8% agarose (Invitrogen) gel, extracted with a QIAquick Gel Extraction kit (Qiagen), purified further with a QIAquick PCR Purification kit (Qiagen), and then sequenced directly using the transposon-specific primer by the Department of Biochemistry DNA Sequencing Facility at the University of Cambridge.

The sequence data were examined using FinchTV v 1.4.0 (Geospiza), then used as queries in blastn searches against a database containing the SL1344 genome, together with the sequences of the Mu and Tn5 transposons. For each mutant, the transposon could be identified as Mu or Tn5 based on which of those sequences gave a higher scoring BLAST hit. The position of the highest-scoring alignment with the SL1344 genome allowed the insertion location and orientation of the transposon to be determined.

### Investigation of attenuation in defined deletion mutant strains

Defined deletion mutants of *S*. Typhimurium were constructed by a modification of the ET-cloning procedure [21]. Genes to be deleted were replaced with a kanamycin resistance cassette from pUC4Kan (Amersham). PCR was used to amplify the antibiotic resistance cassette with 5′ and 3′ 60 bp homology arms complementary to the flanking regions of the gene to be deleted. PCR products were electroporated into *S*. Typhimurium LB5010 [22] containing the plasmid pBADλred expressing the phage lambda genes *exo*, *bet* and *gam* under an inducible arabinose promoter, having induced these cells with 0.2% (w/v, final concentration) arabinose (Sigma-Aldrich) prior to making them electrocompetent. Candidate mutant colonies were selected on LB agar plates supplemented with 25 µg/ml kanamycin. Verification of allelic replacement was carried out by a test PCR using primers designed to regions 150 bp upstream and downstream of the gene of interest. The PCR products were then TA-cloned into pCR®2.1-TOPO (Invitrogen-Life Technologies) following the manufacturer's instructions, and the resultant plasmids were sequenced to confirm the DNA sequence at the junction of the antibiotic resistance cassette and the disrupted gene. The mutations generated in *S.* Typhimurium LB5010 were transduced using bacteriophage P22 [23] into strain SL1344 for *in vivo* virulence studies [24]. Transductants were selected on LB agar supplemented with 25 µg/ml kanamycin and were screened for agglutination with anti-O4 serotype-specific antiserum (Remel Europe Ltd.) in addition to verifying the presence of the mutation by PCR and sequencing, as described above.

Six- to eight-week-old BALB/c mice (Harlan) were used for infection studies using the individual defined deletion mutants. Bacteria were inoculated into L-Broth, supplemented with 25 µg/ml kanamycin if appropriate, and left to stand overnight at 37°C. Bacteria were harvested and re-suspended in PBS and adjusted as appropriate (usually 5×10^3^ CFU ml^−1^ for i.v. infections) and the viable count of the inoculum was confirmed by plating serial dilutions on LB agar. Mice were inoculated with 0.2 ml of the appropriate dilution of the bacterial suspension via the tail vein. Three or four mice per group were killed by cervical dislocation at each time-point post-infection. The spleens and livers of infected mice were removed and placed in 10 ml sterile distilled water and homogenised using a Stomacher® 80 Lab System (Seward). Viable mutants were quantified by plating various dilutions of homogenised organs in LB agar.

For protection experiments, six- to eight-week old BALB/c mice (Harlan) were immunised i.v. with 1.0×10^5^ CFU per mouse of the defined deletion mutant, grown as above, and 4 months later challenged i.v. with 10^4^ CFU per mouse of the virulent parent strain, SL1344. One day prior to the challenge two mice per group were culled and spleens and livers were plated on LB agar to assess the pre-challenge bacterial counts in the organs. When no bacteria were present in these pre-challenge counts, the challenge experiment was undertaken. Non-immunised, age-matched mice were challenged with the same dose of virulent bacteria at the same time as the immunised mice. Three to eight mice per group were killed at each time-point post-challenge and organs plated for viable counts of bacteria as above.

## Results

### Identification of transposon insertion sites using TMDH

**Figure 4:**
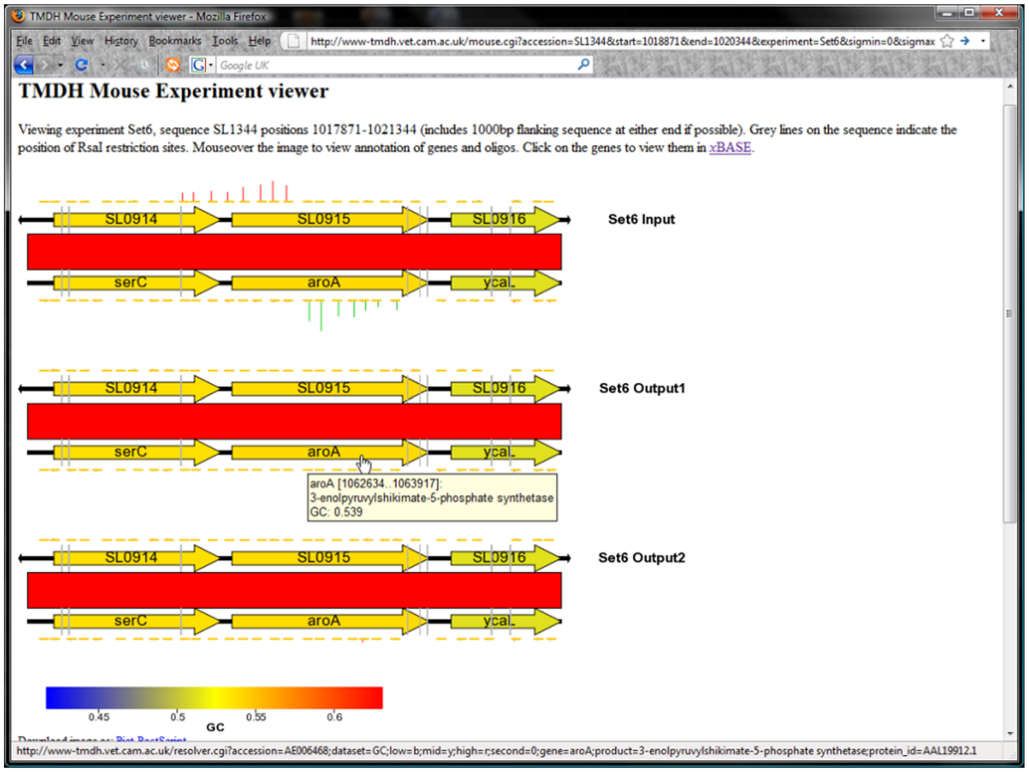
Example of TMDH data displayed using the online database. The database is available at http://www-tmdh.vet.cam.ac.uk.

A list of the transposon mutants identified from the microarray data, together with normalised log_2_-fold change (attenuation) scores and P-values is shown in Table S1. In the table and below, all SL1344 genes are referred to using the name of their LT2 homologue. The list is also available as an online database at http://www-tmdh.vet.cam.ac.uk. This database includes a facility to inspect manually the normalised microarray data and its relationship to the genome sequences of *S.* Typhimurium SL1344 and LT2 (see Figure 4). This provides a powerful tool to confirm the position of transposons determined by the automated algorithm.

**Figure 5:**
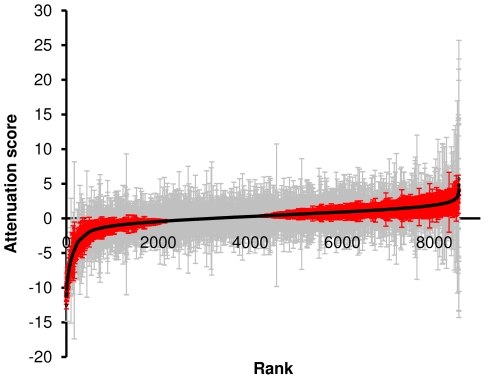
Curve showing the ordered attenuation scores for the 8533 mutants identified by TMDH. Error bars indicate 95% confidence intervals. Red error bars indicate mutants that significantly deviated from 0 (P<0.05), grey indicates genes that did not significantly change in the output.

A graphical representation of the distribution of attenuation scores is shown in Figure 5. The majority of transposon insertions do not affect virulence in this mouse model and show an attenuation score not significantly different from 0. Of the mutants that significantly differ between the input and output pools, the majority are attenuated and under-represented in the output, with an attenuation score of <0. We infer that these mutants have a transposon that disrupts a gene important for infection. A few mutants are over-represented in the output pool relative to the input, suggesting that they may have a mutation that leads to an increase in competitiveness during the infection process. The TMDH analysis procedure allows the positions of transposons to be determined to within a region of around 200 bp. From a total of 10368 mutants, the position of the transposon insertion could be identified using TMDH for 8533. Of these, 6108 could be unambiguously mapped to 2824 different *S.* Typhimurium genes.

### Verification of transposon insertion sites predicted by TMDH

To support the accuracy of the algorithm used to predict transposon insertion sites from the microarray data, sequence data from the regions flanking 50 transposons were obtained (see Methods). 13 of these were selected for sequencing as there was a clear indication of the location of the transposon from the microarray data. This set was chosen to include several mutants that appeared to be attenuated *in vivo*. The other 37 mutants chosen for sequencing were selected randomly from set 6. BLAST searches against the SL1344 genome using the obtained sequence data allowed the position and orientation of the transposon to be accurately determined. The results of this analysis are shown in Table S2. From the 50 transposons investigated, the positions of 46 were within the range predicted by the TMDH analysis. The remaining 4 were situated in positions that did not allow their detection using TMDH, due to the distribution of *Rsa*I restrictions sites and microarray probes (see Discussion).

### Investigation of defined deletion mutants of targets selected by TMDH

**Figure 6:**
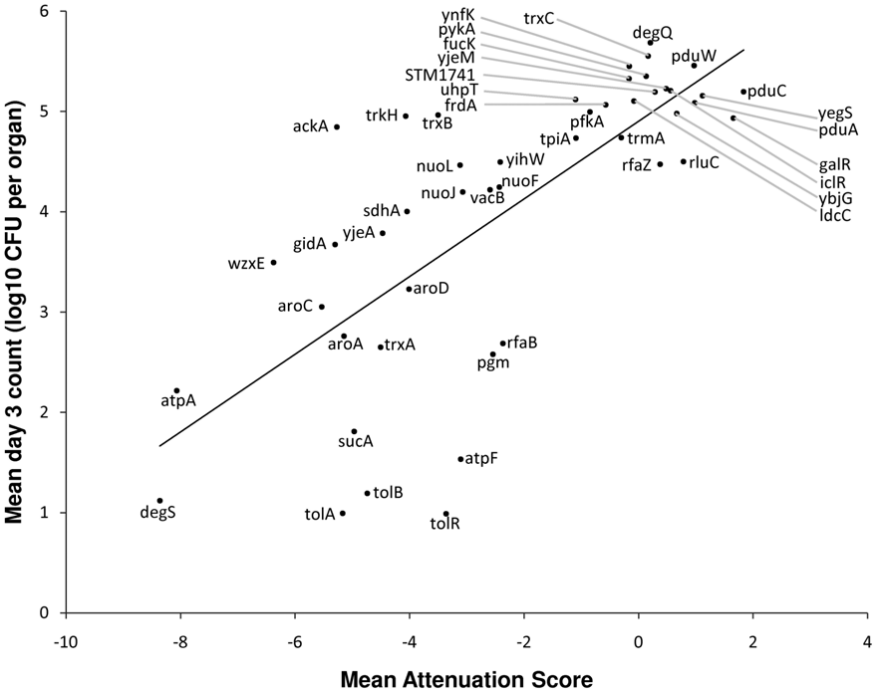
Plot of mean colony counts for individual defined mutant infections against mean TMDH attenuation score. Colony counts were obtained 3 days after infection with defined deletion mutants (10^3^ CFU per mouse). Mean values were calculated for at least three mice per mutant, and compared with the mean TMDH attenuation score for the same gene. The plot shows a significant correlation, demonstrating that TMDH is an effective screen for identifying genes that are important for infection.

Defined deletion mutants were constructed in SL1344 for 47 different genes. These genes were selected to include a representative range of attenuation scores. The *in vivo* growth of each of these was assessed by i.v. infection of BALB/c mice, and compared to infection with the parental SL1344 as a control (see Table S3). Figure 6 shows a plot of the mean bacterial viable counts in the mouse organs on day 3 post-infection, as a function of the mean attenuation score for all the transposon mutants that were unambiguously identified as being within that gene. This plot shows a significant correlation (R^2^ = 0.54, P = 1.7×10^−9^).

### Immunisation with attenuated mutants identified by TMDH and protection against challenge with the virulent parent strain

**Figure 7:**
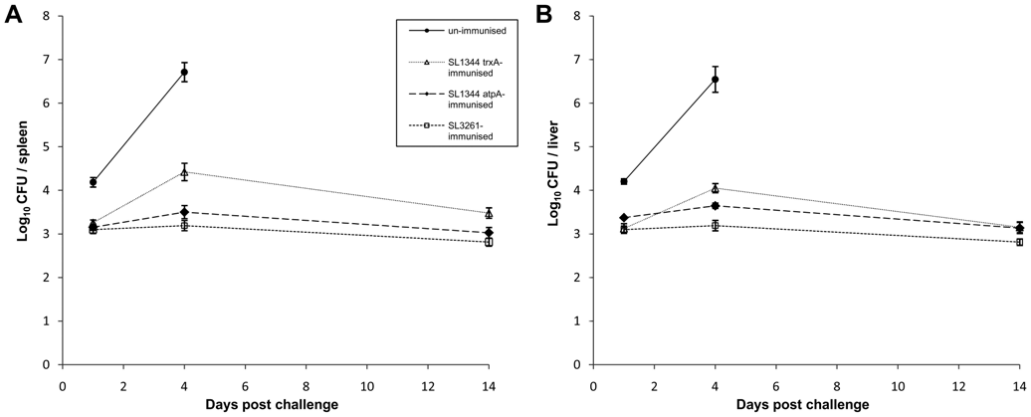
Use of attenuated mutants identified by TMDH as live vaccines. Growth curves for intravenous challenge of mice with SL1344 (10^4^ CFU per mouse), 4 months after intravenous immunisation with SL1344 *trxA*, SL1344 *atpA* or SL3261 (10^5^ CFU per mouse). Bacterial load in (A) spleens and (B) livers of immunised and age-matched un-immunised mice is shown as mean log_10_ CFU per organ. Error bars indicate standard error (n = 3–8).

SL1344 *trxA* and SL1344 *atpA* were chosen from the TMDH screen as live-attenuated vaccine candidates. BALB/c mice were immunised i.v. with 1.0×10^5^ CFU per mouse of SL1344 *trxA* or SL1344 *atpA*. SL3261, an *aroA* mutant of SL1344 which is a well-characterised live-attenuated vaccine strain [24], was used as a control. After 4 months, these mice and age-matched, un-immunised controls were challenged with an i.v. dose of 1×10^4^ CFU SL1344 per mouse. Figure 7 shows bacterial loads in spleens and livers following this intravenous challenge with SL1344. The bacterial counts are represented as the geometric means and standard errors of one representative experiment from two with similar results. Immunisation with SL1344 *trxA* and SL1344 *atpA* resulted in protection of mice against i.v. challenge with the virulent parent strain, as shown by the fact that the viable counts in the organs of immunised animals were lower by several fold than the viable counts in the control un-immunised animals. Infections in un-immunised mice were only allowed to proceed to day 4 before mice were too ill to survive and were culled. In contrast, immunised mice were still well 14 days after challenge with SL1344 showing that *atpA* and *trxA* mutants when delivered intravenously can be used as live-attenuated vaccine strains to protect against a subsequent intravenous challenge. We also investigated the potential of a *tolA* mutant to protect against virulent challenge. *tolA* was selected from the TMDH screen, and encodes part of the Tol-Pal complex in the inner membrane of Gram negative bacteria. SL1344 *tolA* was attenuated in mice via the oral and i.v. routes and i.v. immunisation with SL1344 *tolA* provided significant protection against subsequent challenge with SL1344, delivered i.v or orally [25].

## Discussion

### Comparative *in vivo* TMDH

We have developed TMDH, a microarray-based screen that exploits customised transposons with outward-facing promoters [13] to allow identification of the disrupted genes in pools of transposon mutants [12]. High-density tiling microarrays and a novel bioinformatic algorithm are used to locate the site of transposon insertion with high resolution. This technology is generic with many potential applications. One possibility is the use of large transposon pools to saturate potential sites of insertion, and hence infer genes essential for survival and replication as those which do not contain transposons [26].

An alternative application is comparative *in vivo* TMDH, which exploits the TMDH method to compare a pool of mutants harvested from an infection model with the same pool grown *in vitro*, to identify mutants that are attenuated *in vivo*. Again this is a generic technology and could be applied to the study of any bacterial pathogen for which a genome sequence, a suitable transposon and a reproducible model of infection are available. In the work presented here we have used TMDH to investigate genes important for *S.* Typhimurium SL1344 infection of BALB/c mice.

### Comparison of TMDH with earlier microarray-based methods

There are several previous examples of the use of DNA microarrays to investigate the distribution of transposon insertions in bacteria. The majority of these employ large PCR-product microarrays, with a small number of probes for each gene [9], [10], [27]–[31]. The use of high density tiling microarrays offers a significant improvement over these methods, allowing sub-genic resolution of individual transposon insertion sites. An additional advantage of tiling arrays derives from having probe coverage of the entire genome. This removes the reliance on annotated genome features, reduces the possibility that transposons will be in regions not covered by the microarray, and allows intergenic regions that may be of interest to be determined. One previous study has reported the use of genome tiling microarrays to identify the position of transposons within a bacterial genome [32]. However, the analysis procedure used in that study was a straightforward examination of the microarray signals using arbitrary signal-strength cut-offs and manual inspection. This sort of analysis would not be suitable for interrogation of large pools of mutants such as those screened in the current work. Our analysis method is more sophisticated, allowing an unbiased quantitative measure of the relative fitness of each transposon mutant to be determined.

### Prediction and verification of sites of transposon insertion

In total, 10368 transposon mutants were obtained and investigated using TMDH. From these, 8533 (82.3%) putative insertion sites were identified during the automated microarray analysis. The remainder includes transposons that could not be located as they were inserted in regions of the SL1344 genome not covered by the microarray (including repetitive regions and plasmids or strain-specific islands not present in LT2). Other undetected transposons include those that inserted within small *Rsa*I restriction fragments, and some that could not be unambiguously located due to the presence of two or more transposons in the same orientation within the same restriction fragment. It would be possible to increase the recovery rate by repeating the TMDH procedure using additional restriction enzymes and alternative microarray designs.

The locations of the transposon insertion sites were independently verified by obtaining DNA sequence data from the regions flanking the insert for 37 randomly selected mutants. BLAST searches of the SL1344 genome using the sequence data allowed the location and orientation of each of the transposons to be unambiguously determined. 33 (89.1%) of these were in positions within the range identified by the automated TMDH analysis. The analysis of the remaining 4 insertion events highlights some of the limitations inherent in the method: two were incorrectly identified due to the presence of additional transposons in the same orientation within the same restriction fragment, one was not identified as the insertion was within a small restriction fragment, and one gave a low signal that did not exceed the threshold value used to filter false positives from the dataset. Nevertheless, the correct location of a high percentage of the transposons suggests that the technology is suitable for use as a screening method.

Additional support was obtained by PCR amplification of the regions flanking 13 transposons, using a transposon-specific primer and one designed to be adjacent to the insertion position predicted by the automated analysis. Again, sequence data were obtained using a transposon-specific primer and the position of the transposon relative to the SL1344 genome was determined using BLAST. For this set, all 13 transposons were located within the range predicted by the automated analysis.

### Identification of attenuated *S.* Typhimurium SL1344 mutants

The TMDH analysis procedure distils the microarray data into a form equivalent to a standard expression microarray, and allows the direct comparison of the input and output signals to give a fold-change. This is expressed as a log_2_ value and referred to as an “attenuation score”. Negative values indicate that the mutant is attenuated, while positive values suggest that the mutant has a competitive advantage *in vivo*. Use of replicate mice additionally allows the estimation of a P-value. The data for all 8533 mutants are available in Table S1, ordered by attenuation score.

**Figure 8:**
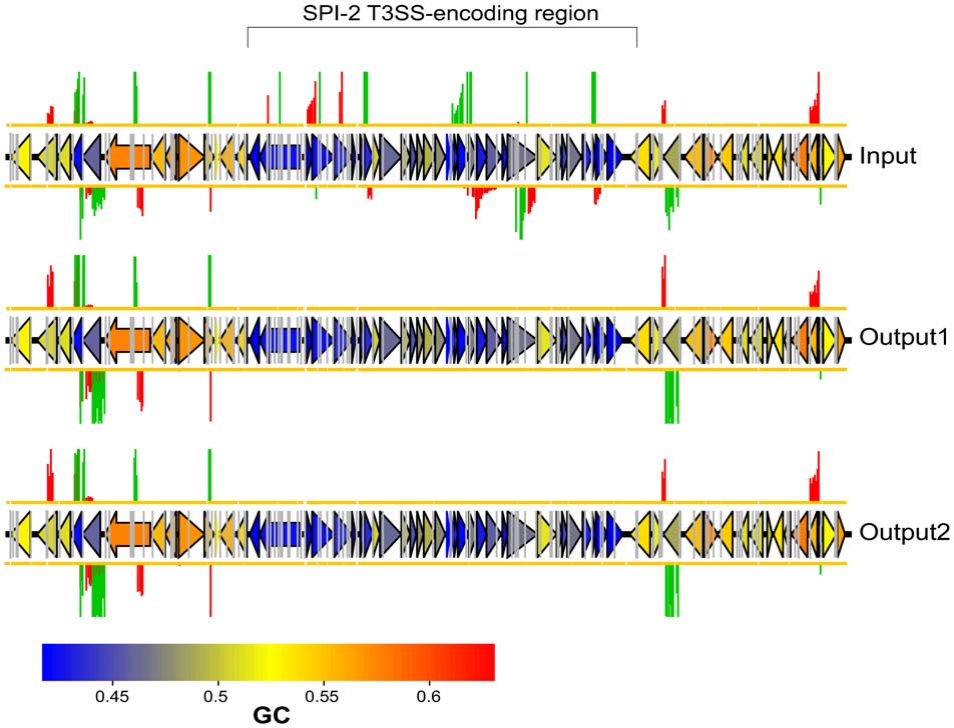
TMDH data obtained from array set 6 for SPI-2 and the surrounding regions. Genes are coloured according to their GC content as indicated by the scale bar. The region of SPI-2 that encodes a type-III secretion system can be identified by its low GC content. The TMDH signals within this region are abolished in the output datasets, indicating that transposon insertions in this region are attenuating. Insertions in the flanking regions give comparable signals in the input and output datasets.

**Figure 9:**
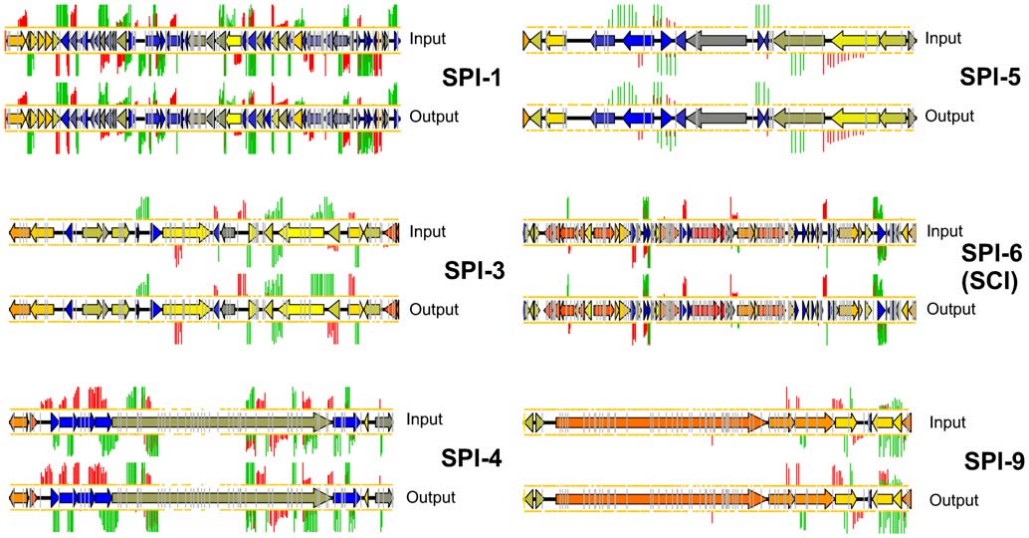
TMDH data obtained for SPI-1, 3, 4, 5, 6 and 9. Transposon inserts within all of these regions result in similar microarray signals in the input and output datasets, suggesting that these regions are not important for infection in our model.

Importantly, the top (most attenuated) end of the list includes many genes that have well established roles in virulence. These include numerous genes that encode structural components of the SPI-2 T3SS or associated regulators, chaperones and secreted effector proteins. The requirement of this system for *S.* Typhimurium infection and persistence is well established [4], and virtually all the transposon mutants within this region are highly attenuated (see Figure 8). Interestingly, transposon insertions within the other SPI regions present in *S.* Typhimurium are not identified as strongly attenuating in our screen (see Figure 9) – this is probably a consequence of the chosen mode of infection (i.v.) and the use of the mouse as the model species.

Beside SPI-2, other well established virulence-related genes that are identified in our dataset include: the aromatic amino acid biosynthesis (or pre-chorismate) pathway genes *aroA*, *aroC* and *aroD*, mutants of which are prototype live attenuated *Salmonella* vaccines [24],[33],[34]; the purine biosynthesis genes *purA*, *purD*, *purF*, *purG*, *purH*, *guaA* and *guaB* [3],[35],[36]; LPS core biosynthesis genes [37]; O-antigen biosynthesis genes [38]; and the virulence plasmid *spv* genes [39]. Presumably because of the requirement for the *spv* operon, plasmid partitioning is required for virulence, and both *parA* and *parB* mutants are attenuated. The latter is surprising since *parB* was described as being dispensable for partitioning in pSLT [40].

As might be expected, bacteria with mutations in many genes previously associated with resistance to acid, high temperature and oxidative stress are unable to survive *in vivo*. Some of these are known to be involved in virulence, including *slyA* [41],[42], *htrA* (*degP*) and *degS* [21],[43],[44]. Other genes that have been associated with stress responses but not previously demonstrated to be attenuated *in vivo* include the tRNA base modification gene *miaA* [45]. The fatty acid biosynthesis gene *fabF* (*atrB*) is associated with the acid tolerance response in *S.* Typhimurium LT2, but previous investigation of a mutant in the SL1344 background did not find any significant effect on acid tolerance or virulence [46]. In contrast, our results suggest that transposon insertions within this gene are attenuating. This may indicate that the *fabF* mutation leads to a mild effect on virulence that is only evident in competitive assays. DNA recombination and repair is also important, with our data confirming the importance of *dam* [47], *recA* [48] and *recG* [49] for virulence. Mutations in the genes *recD*, and to a lesser extent *recF*, *recJ* and *recQ* also appear to be attenuating from our data, however previous studies have not found *recD*, *recF* and *recJ* mutants to be attenuated during individual infection assays [50],[51].

Other systems that are important for infection and persistence in our model include carbon metabolism, with attenuating mutants found in a number of genes involved in glycolysis (*pgm*, *ptsG*, *crr* and *tpiA*), the TCA cycle (*sucABC* and *sdhABC*), mannose utilisation (*mtlA*, *mtlD*, *rfbM* and *manA*) and oxidative phosphorylation (the *nuo* locus, *atpABFI*, *cyoAB* and *cydA* [52]). Zinc transport (*znuABC*) [53] and phosphate transport (*pstABCS*) are also important for virulence, as is pyrimidine metabolism (*pyrBCDE*, and *carAB*). Mutants of two genes involved in the thioredoxin system, *trxA* and *trxB*, are identified as attenuating by TMDH. Of these *trxA* is known to be important for infection of mice [54]. *trxB* mutants are not attenuated in individual infections [54] but do show reduced intracellular proliferation, which may account for their attenuation in the TMDH competitive infection screen.

Genes identified in our screen that had not previously been associated with *Salmonella* virulence include components of the Tol-Pal system that contributes to membrane stability (*tolA* and *tolB*) [25]; *yqiC*, which encodes a putative cytoplasmic protein with no functionally characterised homologues; the putative regulator encoded by STM4030; *ychK*, which encodes a patatin-like lipolytic enzyme; and *ybjT*, which encodes a putative nucleoside-diphosphate-sugar epimerase. Interestingly, the patatin-like ExoU is a type III-secreted cytotoxin and virulence factor of *Pseudomonas aeruginosa* [55], and the *E. coli* O157:H7 homologue of *ybjT* is induced during human infection [56].

Although the TMDH screen is primarily intended to identify attenuating mutants, it also may identify mutants that perform better *in vivo* than *in vitro*, with attenuation scores significantly greater than 0. The genes disrupted in these “hypercompetitive” mutants may impair the infection process in wild-type strains. There are fewer examples of such mutants in our dataset than attenuating mutants (see Figure 5) – intuitively it is easier to see how the infection process may be disrupted than enhanced – and many of the genes at this end of the list have poor P-values. Nevertheless a few plausible candidate hypercompetitive mutants are identified. These include several genes involved in flagellar biosynthesis (*flgCEFKI*, *flhB* and *fliFHIJKRT*), mutants of which are known to display enhanced virulence [57]; the global transcriptional regulator gene *fnr*, mutants of which show enhanced entry into and proliferation within HEp-2 epithelial cells [58]; *dsdA*, which encodes a positive regulator of D-serine deaminase and which when mutated enhances the ability of uropathogenic *E. coli* to infect the bladder and kidneys of mice [59]; and *araH*, the loss of which is associated with virulence in *Burkholderia mallei* [60]. There are also several putative hypercompetitive mutants associated with Type II secretion (*hofQ*, *hofC*, *hopD* and *ppdA*).

### Validation of targets selected by TMDH

47 of the genes identified in the TMDH screen were subjected to further investigation by generating defined deletion mutants and performing single mutant infection assays. The set of 47 genes was chosen to reflect a range of attenuation scores, and to include a number of potential live vaccine candidates. It should be noted that some mutants may be attenuated in parallel infection screens such as TMDH due to their inability to compete with the other mutants in the pool, but not show any evidence of attenuation during a single mutant infection. The reverse is also possible, since a mutant may be able to overcome its deficiency in the presence of other genotypes, for example through the uptake of a compound secreted by the other bacteria. Also, within the TMDH screen, different transposon mutants within the same gene are not necessarily comparable. The transposon may be inserted in a different position within the gene, or a mutant may perform differently in the context of a different pool of mutants. Despite these caveats, Figure 6 demonstrates a significant correlation between the average attenuation score and the average log_10_ colony counts from day 3 of the single mutant infection experiments. This indicates that TMDH attenuation scores accurately reflect the levels of attenuation seen when defined deletion mutants are generated and investigated individually. TMDH therefore represents an effective screen for genes that are important for infection.

Some of the attenuating mutants selected by TMDH were investigated further for their ability to elicit an immune response that would be protective against subsequent challenge with a wild type strain. *atpA* and *trxA* defined deletion mutants protected against wild-type challenge (Figure 7) and further details of similar experiments for another promising candidate, *tolA*, have recently been published [25]. This demonstrates the power of TMDH as a screen for identifying mutants that act as novel live-attenuated vaccine strains.

## Supporting Information

Table S1 TMDH attenuation scores for 8533 transposon mutants. Predicted transposon locations, attenuation scores and P-values for 8533 transposon insertions identified using TMDH. The list is ordered by attenuation score.
(10.39 MB XLS)

Table S2 Transposon locations confirmed by sequencing. Transposon insertion locations predicted by TMDH and confirmed by sequencing for 37 randomly chosen mutants from Set6, and 13 mutants chosen from Set1 and Set3 based on the microarray data.
(0.03 MB XLS)

Table S3 *In vivo* attenuation of defined deletion mutants. Mean day 3 colony counts per liver for mice infected i.v. with wild type SL1344 or defined deletion mutants. P-value indicates probability that the mean day 3 count obtained for the mutant differs from that of wild type SL1344, as determined using an unpaired Student's t-test.
(0.03 MB XLS)
